# Investigation of a Mobile Health Texting Tool for Embedding Patient-Reported Data Into Diabetes Management (i-Matter): Development and Usability Study

**DOI:** 10.2196/18554

**Published:** 2020-08-31

**Authors:** Antoinette Schoenthaler, Jocelyn Cruz, Leydi Payano, Marina Rosado, Kristen Labbe, Chrystal Johnson, Javier Gonzalez, Melissa Patxot, Smit Patel, Eric Leven, Devin Mann

**Affiliations:** 1 NYU Langone Health Department of Population Health Center for Healthful Behavior Change New York, NY United States; 2 NYU Langone Health Medical Center Information Technology Enterprise Project Management Office New York, NY United States; 3 NYU Langone Health Department of Population Health Digital DesignLab New York, NY United States; 4 Rip Road, Inc New York, NY United States; 5 NYU Langone Health Department of Population Health Healthcare Innovation Bridging Research, Informatics and Design Lab New York, NY United States

**Keywords:** patient-reported outcome measures, mobile health, type 2 diabetes

## Abstract

**Background:**

Patient-reported outcomes (PROs) are increasingly being used in the management of type 2 diabetes (T2D) to integrate data from patients’ perspective into clinical care. To date, the majority of PRO tools have lacked patient and provider involvement in their development, thus failing to meet the unique needs of end users, and lack the technical infrastructure to be integrated into the clinic workflow.

**Objective:**

This study aims to apply a systematic, user-centered design approach to develop i-Matter (investigating a mobile health [mHealth] texting tool for embedding patient-reported data into diabetes management), a theory-driven, mobile PRO system for patients with T2D and their primary care providers.

**Methods:**

i-Matter combines text messaging with dynamic data visualizations that can be integrated into electronic health records (EHRs) and personalized patient reports. To build i-Matter, we conducted semistructured group and individual interviews with patients with T2D and providers, a design thinking workshop to refine initial ideas and design the prototype, and user testing sessions of prototypes using a rapid-cycle design (ie, design-test-modify-retest).

**Results:**

Using an iterative user-centered process resulted in the identification of 6 PRO messages that were relevant to patients and providers: medication adherence, dietary behaviors, physical activity, sleep quality, quality of life, and healthy living goals. In user testing, patients recommended improvements to the wording and timing of the PRO text messages to increase clarity and response rates. Patients also recommended including motivational text messages to help sustain engagement with the program. The personalized report was regarded as a key tool for diabetes self-management by patients and providers because it aided in the identification of longitudinal patterns in the PRO data, which increased patient awareness of their need to adopt healthier behaviors. Patients recommended adding individualized tips to the journal on how they can improve their behaviors. Providers preferred having a separate tab built into the EHR that included the personalized report and highlighted key trends in patients’ PRO data over the past 3 months.

**Conclusions:**

PRO tools that capture patients’ well-being and the behavioral aspects of T2D management are important to patients and providers. A clinical trial will test the efficacy of i-Matter in 282 patients with uncontrolled T2D.

**Trial Registration:**

ClinicalTrials.gov NCT03652389; https://clinicaltrials.gov/ct2/show/NCT03652389

## Introduction

### Background

Uncontrolled type 2 diabetes (T2D) is a significant public health problem in the United States, particularly among vulnerable populations (eg, low-income and racial and ethnic minorities) [1,2]. Annually, T2D incurs about US $250 billion in health care costs and lost productivity, representing a significant social and economic burden [3]. Despite recent improvements in the proportion of adults with T2D achieving hemoglobin A_1c_ (HbA_1c_) targets <7%, only 50.9% achieved this level of control [4]. The number of patients who fail to meet these goals is even higher in resource-limited primary care practices—a place where most vulnerable populations receive their care [5,6].

Recognizing the central role patients play in the management of T2D (eg, being aware of its signs and symptoms and engaging in daily self-care behaviors), several national and local organizations have forged initiatives to support the development and use of patient-reported outcomes (PROs) in the evaluation of health and well-being of patients with T2D [7-11]. Measures of PROs are a *standardized and quantifiable* approach that allows for the collection and integration of data on patients’ perspective of their chronic disease into its clinical management [12].

Much of the existing research that incorporates PROs in T2D has been limited to clinical drug trials examining patient tolerance to new treatments [13]. The few practice-based studies conducted in T2D have used long batteries of PRO measures, and patients report PROs only on a single occasion, most often immediately before clinic visits [14,15]. Such reporting introduces a recall bias because patients are asked to approximate changes in their symptoms and behaviors over several months. To address these challenges, a growing number of studies are utilizing mobile health (mHealth) platforms that enable real-time data collection to facilitate patient self-monitoring outside the clinic environment, enhance patient engagement in their care, and inform provider decision making [16-21].

Systematic reviews of mHealth interventions in patients with T2D have demonstrated positive, short-term benefits on HbA_1c_ levels and self-care behaviors [22-24]. However, these studies have several methodological shortcomings that limit their impact, including small sample sizes (24-180 patients), short study duration (mean 24 weeks), low patient compliance, limited integration with clinical practice, and exclusion of vulnerable populations that would benefit most from mHealth interventions [25]. More importantly, the PROs collected in the mHealth tools are researcher-driven and lack patient and provider involvement in the conceptualization of the intervention*.* As a result, the tools are not customized to address the complex and unique needs and preferences of patients and lack the technical infrastructure to support integration into the clinic workflow.

### Objectives

The i-Matter (investigating an mHealth texting tool for embedding patient-reported data into diabetes management) trial aims to address this gap in the literature by evaluating the efficacy of an innovative mobile PRO system that incorporates patients’ perspective of their disease into the management of T2D in primary care practices. The i-Matter intervention uses text messaging to capture patients’ self-reported PROs in real time, enhances patient engagement through data-driven feedback and motivational messages, and creates dynamic visualizations of the PROs that can be shared in personalized reports and integrated into the clinical workflow. A future randomized controlled trial (RCT) will evaluate the efficacy of the i-Matter intervention versus usual care on reduction in HbA_1c_ and adherence to self-care behaviors at 12 months among 282 patients with uncontrolled T2D who receive care in resource-limited primary care practices. This paper discusses the iterative process of developing, integrating, and user testing the i-Matter intervention in the formative phase of the trial.

## Methods

### Theoretical Framework

The i-Matter intervention is a blend of 2 frameworks: technology acceptance model (TAM) and capability, opportunity, and motivation model of behavior (COM-B). The TAM is based on the theory of reasoned action and asserts that perceptions of usefulness and ease of use directly influence the intention to use a new technology, leading in turn to its adoption [26]. The TAM is considered a *gold standard* for characterizing the adoption and use of new health information technology [27,28]. COM-B is a parsimonious amalgamation of existing theories of behavior change [29], which states that interaction among 3 key components is necessary for successful behavior change: the person needs to feel *capable* (ie, the ability to engage in necessary physical and thought processes) of changing, needs to have the *opportunity* (ie, social and environmental factors) to change, and needs to feel *motivated* (ie, confidence and self-efficacy) to change [29]. The COM-B model has been proven effective for designing programs that help patients with T2D improve adherence to health behaviors [29,30]. The core components of the COM-B model are integrated into the design features of the i-Matter intervention to create a theoretically grounded technology solution (Table 1) [31-33].

**Table 1:** Application of capability, opportunity, and motivation model of behavior theoretical constructs to i-Matter design features.

i-Matter^a^ components	COM-B^b^ constructs	Mechanisms of action	Design features
PRO^c^ assessments	Capability (comprehension) Motivation (habit formation)	Rating PROs on a scale helps patients make more realistic assessments of their symptoms and behaviors Daily ratings increase patients’ awareness of their condition on their quality of life and daily functioning Tracking PROs and observing patterns provides patients with reasons to adhere to their self-management regimen	Daily text message questions Asks patients to complete small doable actions at optimal times
Feedback messages (insights)	Motivation (perceptions of illness and emotional response)	Enables patients to identify changes in PROs that previously went undetected Encourages self-reflection of PRO ratings and their impact on behavior	Data-driven insights based on PRO ratings, such as: Correlational: association between PRO responses Individual: comparisons of PRO responses across weeks
Motivational messages	Motivation Opportunity (perceived support)	Uses text messages to maintain high levels of engagement in the program	Text messages that encourage journaling, such as: Response-based: weekly supportive messages based on PRO responses Activity-based: weekly messages based on response rates to the messages Completion-based: messages based on patient duration in the study
Personalized reports	Opportunity (patient-provider relationship) Capability (comprehension and ability to plan)	Facilitates informed discussions with provider Provides provider with succinct and timely data on patient PROs Motivates patients through the gradual completion of the personalized report, with landscape changes every 4 weeks Enables patients to understand and identify patterns in their PROs and to develop behavioral changes to better manage PROs	Patient reports will include pattern messaging, PRO data visualizations, reflective questions, and tips plus an area for notes Monthly PRO patterns integrated into EHR^d^, available during and between visits

^a^i-Matter: investigating an mHealth texting tool for embedding patient-reported data into diabetes management.

^b^COM-B: capability, opportunity, and motivation model of behavior.

^c^PRO: patient-reported outcome.

^d^EHR: electronic health record.

### Overview of the Study Design

We used the evidence-based user-centered design (UCD) approach to conduct the formative phase of the trial [34-37]. The aims of this phase were to (1) systematically gather and incorporate feedback from patients and providers to develop and refine the i-Matter intervention and (2) optimize the design of the personalized report for patients and providers [38,39]. The formative phase consisted of 3 steps: (1) focus groups and semistructured interviews to *adapt* i-Matter to diverse patient and provider needs, including those of Spanish-speaking patients; (2) a design workshop to understand a *day in the life* of patients with T2D and provider workflow processes to *integrate* i-Matter into clinical practice; and (3) user testing to *evaluate* the usability and acceptability of i-Matter in patients with T2D and optimize the tool’s performance and display of the personalized reports. The primary outcome of this phase was a refined, integrated, and well-tested mobile PRO system for T2D whose efficacy will be evaluated in the clinical trial.

### Study Setting and Population

This study was conducted in a network of primary care practices of New York University Langone Health (NYULH). The practices comprised >1500 ambulatory physicians, nurse practitioners, and physician assistants who care for >800,000 patients in 235 facilities in New York City’s 5 boroughs: Long Island, New Jersey, Westchester County, Putnam County, and Dutchess County. The participating sites include academic practices, many community-based practices, and federally qualified health centers, serving an ethnically diverse population. All primary care practice sites share a single, integrated electronic health record (EHR; Epic).

The target enrollment for the formative phase was 36 patients and 14 providers. To be eligible, patients must (1) have had a diagnosis of T2D for ≥6 months; (2) have had uncontrolled T2D, defined as HbA_1c_ >7%, documented in the EHR at least twice in the past year; (3) be fluent in English or Spanish; (4) be willing to send and receive text messages; and (5) be aged ≥18 years. Patients were excluded if they (1) refused or were unable to provide informed consent; (2) had acute renal failure, end-stage renal disease (ESRD) or evidence of dialysis, renal transplantation, or other ESRD-related services documented in the EHR; (3) were participating in another T2D study; (4) had significant psychiatric comorbidity or reports of substance abuse (as documented in the EHR); (5) were pregnant or planning to become pregnant within 12 months; or (6) planned to discontinue care at the practice within the next 12 months. Providers were eligible if they (1) were a primary care provider (ie, medical doctor, nurse practitioner) practicing at the participating practices and (2) provided care to at least five patients with T2D. The NYULH Institutional Review Board approved this study.

### Recruitment

We used 2 approaches to recruit patients and providers into the formative phase. First, potentially eligible patients were identified through a review of the EHR using the diagnosis-related group codes indicating the presence of T2D and receiving care from a primary care provider at one of NYULH practices. After retrieving a list of potentially eligible patients, research assistants (RAs) reviewed patients’ EHR to determine if the patient met the eligibility criteria. Patients that met these criteria were contacted via telephone to confirm eligibility. During the telephone call, the RA gave patients a description of the study, including their role as participants in the study. If the patient remained interested in participating, they were given the option to either complete the focus groups or interviews in-person in a private room or via a remote session using the secure Webex conferencing platform. Providers were sent emails from the study principal investigators inviting them to provide feedback on the development of an interactive mHealth tool that could help enable patients with T2D to take a more active role in their diabetes management. All patients and providers provided written informed consent before participation in the study.

### Development of the i-Matter Intervention

Table 2 provides an overview of the UCD process used to develop the PROs for i-Matter. A description of each step is also included below.

**Table 2:** Evidence-based user-centered design process for the development of patient-reported outcome text messages.

Steps	Methods	Outputs
1. Adapt	Patient focus groups and provider interviews	Thematic analysis of patient and provider needs, preferences, and barriers and facilitators of tracking PROs^a^ Review of existing validated PRO questionnaires by study team based on thematic analysis Initial list of PROs for i-Matter^b^ comprised individual items extracted from existing questionnaires Reduced list of PROs based on importance rankings from focus group participants
2. Integrate	Design workshop Workflow mapping Problem or opportunity analysis Presentation of PRO list from step 1 EHR^c^ integration	Refined list of PROs Clinic workflow or patient journey maps Essential features of i-Matter system i-Matter^b^ prototype: PRO text messages and personalized report
3. Evaluate	2 rounds of patient user testing sessions Provider interviews	Finalized PROs and personalized report Fully functional i-Matter intervention

^a^PRO: patient-reported outcome.

^b^i-Matter: investigating an mHealth texting tool for embedding patient-reported data into diabetes management.

^c^EHR: electronic health record.

#### Step 1. Focus Groups and Interviews to Adapt i-Matter to Diverse T2D Patients and Primary Care Physician Needs

The goal of the focus groups was to select the PROs that would be integrated into the i-Matter intervention as it relates to patients’ experiences living with T2D. A trained moderator used a semistructured guide to explore (1) patients’ daily experiences living with T2D, (2) the barriers and facilitators to achieving their diabetes-specific goals, (3) descriptions of patient-provider conversations about T2D and goals for HbA_1c_, and (4) interest in sharing PRO data with their provider to support treatment of T2D. A trained bilingual moderator also conducted separate focus groups with Spanish-speaking patients to inform the cultural and linguistic adaptation of i-Matter. Before starting each focus group, all patients completed questions about their comfort with using technology and their interest in using mHealth tools for diabetes care.

A trained moderator conducted semistructured individual interviews with primary care providers at the participating practices. The goal of the interviews was to elicit provider feedback on the clinical relevance of the PROs discussed in the patient focus groups for the management of T2D. The interview guide also explored (1) providers’ level of comfort with PRO data, (2) descriptions of patient-provider discussions about diabetes management, and (3) other important PROs not identified in the patient groups.

Results from the thematic analysis of the focus groups and interviews were used to develop a preliminary list of PROs for inclusion in i-Matter [40]. The PROs were individual items taken from existing validated PRO measures that assess the impact of T2D and its treatments on patients’ psychosocial, physical, and behavioral functioning (eg, emotional distress, treatment and disease burden, adherence to medications, and lifestyle behaviors) [41]. The measures included the Problem Areas in Diabetes Questionnaire, Diabetes Treatment Satisfaction Questionnaire, Treatment Related Impact Measure-Diabetes, Audit of Diabetes-Dependent Quality of Life, Diabetes Impact Management Scale, and Diabetes Distress Screening Scale [42-47]. General items from the National Institutes of Health Patient-Reported Outcome Measurement Information System Global 10 measure, which assesses patients’ physical, social, and emotional functioning [48], were also included on the list of candidate PROs.

The study team then recontacted patients from the focus groups to get their feedback on the candidate list and have them rank the perceived importance of each PRO for management of T2D on a 1 (least important) to 6 (most important) scale. The study team used patients’ ratings in concert with the thematic analysis to narrow the list of PROs that would be presented to participants in the design workshop.

#### Step 2. Integrate i-Matter Into Provider Workflows and Patient Daily Lives

The design workshop comprised patients, providers, academic researchers with expertise in T2D and PROs; the digital health company Rip Road; and staff from the NYULH Medical Center Information Technology (MCIT) department. The design workshop used a UCD protocol adapted from the Agency for Healthcare Research and Quality [49] that sequentially led the group through a variety of activities (eg, story mapping and workflow or patient journey analysis) designed to further refine the i-Matter PRO content, stimulate ideas for the content and layout of the personalized report, discuss ideal workflow integration, and identify potential problems and opportunities to improve i-Matter for patients and providers.

Following steps 1 and 2, the study team collaborated with Rip Road to develop a prototype of i-Matter (ie, the beta version of the text message program and personalized report).

We wrote 2 variations of each PRO question to evaluate the wording and response formats that would yield the highest patient response rates and data quality. On the basis of our previous experiences and best practices for data collection via text message [50], all PRO questions were written so they require short, simple answer choices (eg, 0-10 rating or yes or no response), thereby minimizing the likelihood of missing and/or unanalyzable data that is common with open-ended (free text) response options. We also created 2 versions of the personalized report: a 1-month view and a 3-month view. All text messages and report content were translated to Spanish before user testing.

**Figure 1:**
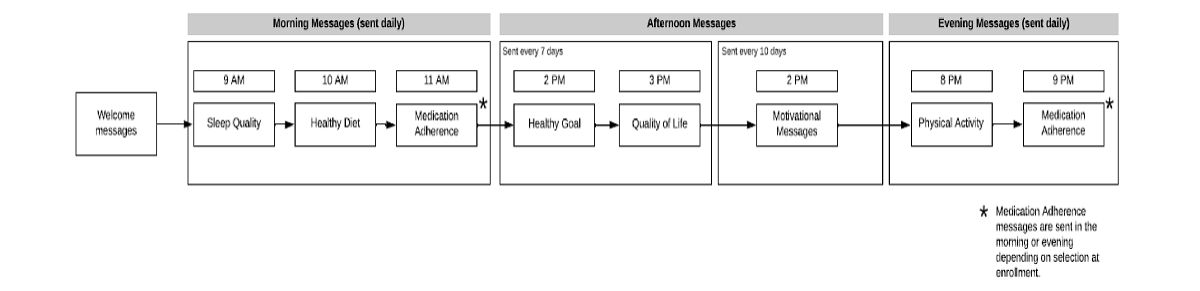
i-Matter study flow.

In addition to prototype development, we created decision rules that would drive the delivery of the text messages and report content. The rules, which were iteratively refined throughout the formative phase, outline the timing and order of the messages, the duration of time patients had to respond to each message (ie, response window), and the conditions that triggered specific motivational text messages and individualized insights displayed on the personalized report (Figure 1). As shown in Table 1, patients receive 3 types of motivational text messages over the course of the study: (1) response-based, (2) activity-based, and (3) completion-based. The personalized report displays 2 types of insights: (1) correlational, which compares associations between 2 different PROs, and (2) individual, which identify trends in patients’ responses to the PROs over the past month (see Multimedia Appendix 1 for example messages).

#### Step 3. User Testing of the i-Matter Prototype

**Figure 2:**
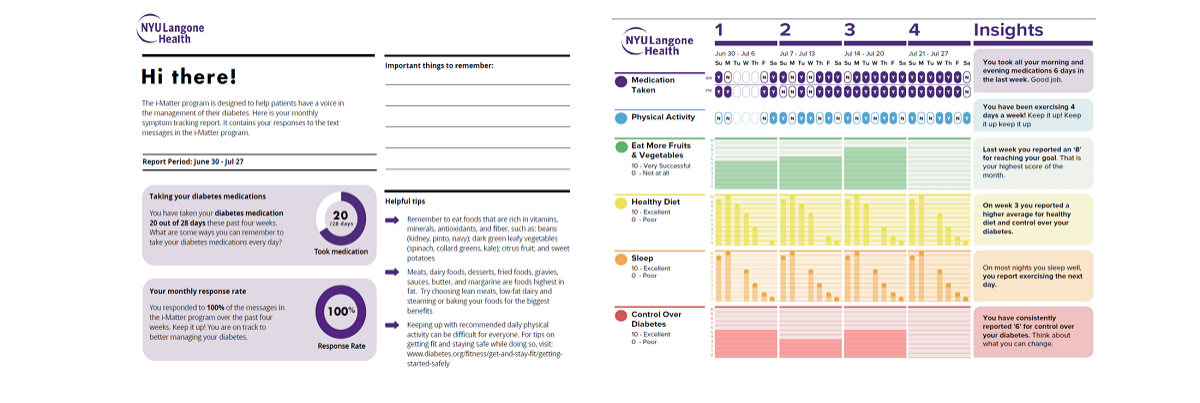
Example of a final personalized report after two rounds of user testing.

User testing was conducted in a purposive sample of patients drawn from the focus groups and those who were naive to the tool (ie, did not participate in previous steps). A rapid-cycle design (ie, design-test-modify-retest in short intervals of time) was used to allow for iterative refinement of the i-Matter prototype between each user test. Patients participated in the user testing sessions for 2 weeks, during which time they received and responded to the PRO questions sent via text message. At the end of the 2-week period, the study team sent patients a copy of the personalized report (Figure 2) and conducted an interview about their experiences. The interviews used a combination of think-aloud techniques and semistructured questions to collect patient feedback on the perceived ease of receiving and responding to the PRO questions; the clarity, timing, and frequency of the messages; and the perceived usefulness of systematically tracking the selected PROs for diabetes self-management. Patients also provided feedback on the personalized report, including the clarity of the data visualizations and data-driven feedback messages (herein called insights), content and layout of the report, and utility of the report for diabetes self-management. In addition to the interview, patients responded to questions derived from the TAM version 3 (TAM3) survey.

We also conducted interviews with providers to elicit their feedback on preferences for visual displays and placement of the report in the EHR and perceived barriers and facilitators to viewing the reports in clinical practice. The primary outcome of this step was the fully functional i-Matter intervention for testing in the RCT.

### Measures

*Participant demographics*: a self-report instrument was used to collect patient sociodemographic data including gender, race or ethnicity, age, annual household income, education level, marital status, employment status, and current HbA_1c_ level.

Patient use of mobile technology: before the focus groups, patients completed a survey created for this study that assessed the frequency of mobile phone use, capabilities of their mobile phones (eg, Wi-Fi connection, Bluetooth, and mobile data plan), the most commonly used functions (eg, text messaging, phone calls, email, and apps), comfort with using their mobile phone to manage T2D, interest in enrolling in a text messaging diabetes program, and challenges to using their mobile phone for diabetes self-management.

*Use behavior:* these data were extracted from the i-Matter platform at the end of the user testing sessions and included the following metrics (described in the analysis section): number of mobile phone inputs, time-on-task, task success, number of missed responses to PRO questions, and number of responses by patients outside the response window.

*TAM3 survey:* following the 2-week user testing period, patients completed questions derived from the well-validated TAM3 survey that assessed the perceived ease of use, usefulness, and quality of i-Matter; the likelihood of using i-Matter in the future and recommending it to others (ie, behavioral intention); and perceived benefits of discussing i-Matter data with providers to help manage their diabetes (ie, communication). The internal consistency of this scale ranged from 0.86 (communication) to 0.94 (perceived usefulness).

### Statistical Analysis

Sample size estimates for the formative phase were based on best practices for maximizing the information power of qualitative research, which recommends beginning with 6 to 8 participants per qualitative method and adding to the sample, as needed [51]. As with previous studies, user testing sessions were scheduled until data saturation was reached [36]. Our previous studies suggested that we would need 2 to 3 cycles of user testing to reach saturation [36].

Focus groups and interviews were audiotaped, translated where necessary, and transcribed verbatim. Both data sources were analyzed using the constant comparative method, in which text was categorized into themes with the use of codes developed iteratively to reflect the data [52,53]. The coding scheme was developed by the study investigators to focus on key themes identified both a priori (eg, from the interview protocols) and those that emerged during the interviews or focus group discussions. A trained qualitative researcher coded the transcripts independently, after which the research team met to discuss the coding and resolve any discrepancies.

After each round of user testing, the study team employed the best practices for instant data analysis of usability data for each PRO [54,55]. Task success was calculated as the percentage of PRO questions that were answered correctly without errors. Time-on-task was calculated as the average amount of time in minutes and seconds that patients took to respond to each PRO question. Mobile phone inputs were calculated as raw counts of PRO questions sent by the i-Matter platform and the number of responses received by patients. Missing data were calculated as the percentage of PRO questions that had no response by patients, and late responses were calculated as the percentage of messages sent by patients that was outside a 1-hour window. In addition, we calculated frequencies for the TAM3 survey questions.

Following the analysis of use data, the research team categorized each issue with usability as either critical (abandon or remove), severe (significant delay or frustration in task completion requiring revision), or cosmetic (minor issue). Each of these issues were mapped onto the interview transcripts and survey responses to provide specific and detailed recommendations for refining i-Matter before proceeding to the next testing session.

## Results

### Step 1. Patient Focus Groups, Provider Interviews, and Ranking of Candidate PROs

We invited 55 patients with T2D (22 male and 33 female) to participate in the focus groups, of which 35 (64%) declined participation, leaving 20 potential participants. Reasons for declining participation included being too busy, limitations owing to other comorbid conditions, personal or family constraints, and lack of interest in participating in the research. Of the 20 people who agreed to participate, 12 (60%) attended one of the focus groups, 1 did not attend owing to a scheduling conflict with work, and 7 stopped responding to the RA’s outreach calls. We held 4 focus groups: 2 for English-speaking patients (n=6) and 2 for Spanish-speaking patients (n=6). Table 3 describes the sociodemographic characteristics of the focus group participants and their comfort with technology.

Analysis of the focus groups identified 4 core themes: (1) patients felt as though their lives were controlled by their blood sugar values; (2) patients’ greatest fear of having T2D were vision loss, kidney failure, or risk of amputation, and avoiding these consequences served as motivators for behavior change; (3) important goals for patients were being in control of their T2D, feeling well, living a long healthy life, and eventually not needing medications for T2D (owing to concerns about the negative long-term effects); and (4) forgetfulness, poor dietary adherence, physical inactivity, tiredness or fatigue, and poor emotional health were viewed as major barriers to keeping blood sugar in control. Patients in the Spanish-speaking focus groups also spoke about God being an important source of strength and motivation to improve their health.

We conducted 6 provider interviews (3/6, 50% female; 4 primary care providers, 1 endocrinologist, and 1 general surgeon and weight management specialist). Analysis of the interviews identified the central theme that providers want PRO data that are specific and actionable and can help them focus the clinic visit on what is most important for their T2D patients’ care. All providers felt that an asset of a program like i-Matter would be having patients systematically track data such as dietary intake and medication adherence that they cannot reliably assess within the time constraints of a clinic visit. All providers liked the idea of showing correlations between PROs being tracked in i-Matter and clinical data that are already stored in the EHR, such as HbA_1c_ values. Providers varied on the importance of tracking patient functional status, quality of life, and psychosocial health, with two-thirds of the providers commenting that it was central to understanding patients’ behaviors, whereas the remaining one-third felt they were *soft symptoms* that may be important for the patient but not for clinical management.

Next, the study team selected individual items from existing PRO measures that best represented themes derived from the focus groups and interviews. This resulted in the selection of items representing 8 categories of PROs: diabetes-related symptoms, quality of life, emotional health (eg, depression, mood, and distress), treatment-related symptoms, treatment satisfaction, diabetes-related functional status, medication adherence, and lifestyle behaviors. Patient ranking of the items further reduced the number of PRO categories to 5: diabetes-related symptoms, quality of life, emotional health, medication adherence, and lifestyle behaviors (Table 4).

**Table 3:** Sociodemographic characteristics and comfort with technology survey responses among focus group participants (n=12).

Sociodemographic characteristics			Values
Age (years), mean (SD)			62.5 (5.6)
HbA_1c_^a^, mean (SD)			7.95 (0.8)
Female, n (%)			8 (67)
Employed, n (%)			4 (33)
Retired, n (%)			4 (33)
Annual income <US $25,000, n (%)			7 (58)
Hispanic, n (%)			7 (58)
**Race, n (%)**			
	White	5 (42)	
	Black	3 (25)	
	Asian	1 (8)	
	Other	4 (25)	
**Education, n (%)**			
	Less than high school	1 (8)	
	High school degree	4 (3)	
	Some college	2 (17)	
	College degree	5 (42)	
**Technology survey, n (%)**			
	Currently uses text messaging	7 (58)	
	Has an unlimited text messaging plan	12 (100)	
	Always has mobile phone with them	9 (75)	
	Comfortable downloading apps on their mobile phone	7 (58)	
	Comfortable receiving and responding to text messages about T2D^b^	8 (67)	
	Interested in using mobile phone to help keep track of T2D	7 (58)	
**Challenges to tracking T2D on mobile phone, n (%)**			
	Cost of receiving messages	2 (17)	
	Unreliable internet access	1 (8)	
	Do not use mobile phone regularly	3 (25)	
	Unsure of benefit	4 (33)	
	Concerns about privacy and security	2 (17)	

^a^HbA_1c_: hemoglobin A_1c_.

^b^T2D: type 2 diabetes.

**Table 4:** Patient ranking of perceived importance of initial list of candidate patient-reported outcomes.

PRO^a^ categories	PRO statements	Mean importance score, range 1 (low) to 6 (high)
Symptom	Over the past week, did you experience tingling or prickling sensations in hands or feet owing to your diabetes?	1.8
Symptom	Over the past week, did you experience dry mouth owing to your diabetes?	3.0
Symptom	Over the past week, how often were you bothered by blurred vision?	3.6
Symptom	Over the past week, how would you rate your level of fatigue owing to your diabetes?	4.3
Symptom	Over the past week, how often did you experience increased thirst and frequent urination owing to your diabetes?	4.1
Emotional health	Over the past week, how often were you been bothered by emotional problems such as feeling anxious, depressed, or irritable owing to your diabetes?	4.2
Emotional health	How often over the past month, did you feel overwhelmed by the demands of living with diabetes?	2.75
Lifestyle behavior	On average, how many days did you participate in at least 30 min of physical activity over the past 7 days?	3.13^b^
Lifestyle behavior	Over the past week, how often did you eat (favorite unhealthy food)?	4.6
Lifestyle behavior	Over the past week, how often did you eat (favorite healthy food)?	2.25
Lifestyle behavior	How would you rate your sleep quality over the past 7 days?	4.8
Quality of life	I was able to keep my blood sugar in good control today.	4.6
Medication adherence	Over the past week, how often were you able to take your diabetes medication on time?	4.9
Medication adherence	How many days did you miss or skip at least one dose of your diabetes medication over the past 7 days?	2.9

^a^PRO: patient-reported outcome.

^b^Despite the lower score, physical activity was added as a PRO after review of transcripts and notes from patient and provider interviews.

### Step 2. Design Workshop

A total of 17 stakeholders participated in the design workshop. The following themes emerged when the group discussed the candidate list of PROs: (1) PROs should show variability in patients’ responses over time and be actionable by both patients and providers, (2) PROs should be taken from validated questionnaires to increase provider confidence in the data patients report and be comparable with HbA_1c_ levels, (3) choosing fewer PROs would help increase patient response rates and reduce the burden on providers to view the data, (4) tracking PROs that focus on adherence to lifestyle behaviors were most appealing to patients, and (5) PRO content should be general (eg, “how are you feeling today?”) as opposed to diabetes-specific (“how much does diabetes interfere with your life?”). The group reasoned that questions that were too specific may not be relevant to all patients and could lead to disengagement or missing data. Alternatively, a broader question could be used as a way to show care for patients’ overall well-being and as an entry point for more diabetes-specific questions that may uncover new or different concerns the provider should be aware of.

On the basis of these discussions, the group generated several ideas for potential visualizations of PRO data. These included defining a threshold that patients’ data can fall above or below and depicting it in a way that makes it easily detectible and actionable, using bar graphs to show directionality, including icons or coloring schemes in addition to PRO labels that enhance the readability of the report, and including summary data in percentages or raw numbers to show the patient’s progress over time.

Applying the findings from steps 1 and 2, the study team reduced the number of PRO categories to 4. Diabetes-related fatigue (symptom category) was removed from the list because providers viewed it as too nebulous and not actionable, whereas patients felt sleep quality was a more meaningful PRO for their diabetes management. In addition, physical activity was added to the lifestyle category because many patients felt that physical inactivity was a major contributor to weight gain and poor diabetes control.

### Step 3. User Testing of the i-Matter Prototype

#### Patients: Text Messages

We completed 2 rounds of user testing with patients: 7 patients completed the first round of testing (1 Spanish-speaking), and 3 patients completed the second round. Table 5 presents the results of the use behavior data for both rounds of user testing. The i-Matter platform sent 325 messages, and patients sent 256 responses (78.7%). The most common reason for invalid messages was the response being sent in the wrong format (eg, sending free-text responses instead of a numerical response). The most common reasons for missing messages included problems with message filtering by the mobile carrier (which has been resolved by changing to short code messages), being busy during the response window, and not having their phone during the time the messages were sent. For the Spanish-speaking patient, the average response rate was 67.3 min (range 0-209.1 min). Overall, 59.7% (153/256) of the messages were answered within an hour, of which all (256/256, 100%) were answered within 1 min.

In the second round of user testing, the message protocol was modified to address the suboptimal percentage of missed responses. For example, to address the wide range of response times seen in the first round of testing (range 0-661.6 min), we restricted the patients’ ability to respond to the morning and evening PRO questions to a 1-hour window (based on the median response time). Overall, the i-Matter platform sent 222 messages and received 188 responses (84.6%) from patients. The most frequently missed message was sleep quality (77/188, 40.9% of missed messages). The most common reason for an invalid message was the patient responding to a question outside the 1-hour response window.

In qualitative interviews, patients in both rounds of user testing described the program as easy to use, not intrusive to their daily life, and helpful for managing their T2D. Similar findings were seen in the TAM3 survey responses (Table 6). Patients also liked the consistency in message timing because it helped them build a habit to respond (“it becomes second nature”). Several patients commented that they felt as though a person was sending the messages to check up on them. Patients also felt that the number of messages sent was adequate, with 2 people commenting, “No number is too many because they want to get better.” There were no differences in qualitative feedback or TAM3 responses between the English- and Spanish-speaking patients.

**Table 5:** Patient text messaging use behavior during user testing.

User behaviors	User testing round 1 (n=7)	User testing round 2 (n=3)
Time-on-task	44 min (range 0-661.6)	20 min (range 0.08-30)
Task success (messages), n (%)	232 (90.6)	175 (93.1)
Missed responses, n (%)	100 (39.2)	28 (15.0)
Late responses, n (%)	49 (19.3)	14 (7.5)
Invalid responses, n (%)	24 (9.4)	13 (6.9)

**Table 6:** Response to technology acceptance model version 3 survey questions.

Questions		Proportion of patients agreeing with statement, n (%)
**PRO^a^** **(n=7)**		
	I would definitely use the i-Matter program in the future	5 (71)
	The PRO questions are very helpful for managing T2D^b^	6 (86)
	Receiving and responding to PRO questions was easy	7 (100)
	I responded to the PRO questions all the time	5 (71)
	I would recommend i-Matter to friends and family	7 (100)
	My provider would be more effective managing T2D with my PRO data	5 (71)
	Overall, the i-Matter program is great or excellent	6 (86)
**Personalized report (n=9)**		
	I would definitely use the personalized report in the future	8 (89)
	The personalized report is very helpful for managing T2D	7 (78)
	The personalized report is easy to use	5 (56)
	I would share the personalized report with friends or family	5 (56)
	Showing my provider the personalized journal would help make clinic visits more effective	7 (78)
	The charts and images are great	6 (67)
	Overall, the personalized report is great	6 (67)

^a^PRO: patient-reported outcome.

^b^T2D: type 2 diabetes.

As shown in Table 7, patients recommended improvements to the wording and timing of several of the PROs (eg, sending the sleep message at 9 AM rather than 7 AM), which is reflective of the use data. Patients also recommended including motivational messages to help sustain engagement with the program. After examining the data, the study team decided to remove the emotional health PRO (ie, labeled as critical). This was owing to the lack of variability in the use data (206/256, 80.6% of responses were 0-1 on a 10-point scale) and feedback from patients in the interviews that the PRO was not relevant to the management of their T2D.

**Table 7:** Recommended changes to patient-reported outcome text messages from user testing sessions.

PRO^a^ categories and original messages		Original timing	Revised message	Revised timing
**Medication adherence**				
	Have you taken all of your diabetes medications as prescribed today?	Daily at 7 AM	Retain as is	Allow patients to decide if they want the message in the AM or PM, or both (11 AM and 9 PM)
**Lifestyle**				
	Reply with 1-4 to track ONE healthy living goal: 1=Lose weight 2=Eat more fruits and vegetables 3=Eat less sweets and carbohydrates 4=Have better portion control	Daily at 7 AM	Retain for all patients. Separate less carbs and sweets to 2 separate goals	Changes so patients choose healthy goal at baseline visit (with option to change goal every 3 months)
	How successful were you in achieving your goal to (custom text healthy goal) yesterday? Response: 0 (not at all) to 10 (very successful)	Daily at 2 PM	How successful were you in achieving your goal to (custom text healthy goal) this *past week?*^b^	Change timing to weekly at 2 PM
	In general, how healthy your overall diet was today?	Daily at 7 AM	Retain message but change timing to assessing overall diet *yesterday*^b^	Change to daily at 10 AM
	Rate your sleep quality last night. Think how easily you fell asleep, how often you woke up and if sleep was refreshing. Response: 0 (poor) to 10 (excellent)	Daily at 7 AM	Reply with the number that best describes how well you slept last night	Change to daily at 9 AM
	How many days in the past week did you do any physical activities like brisk walking where you breathed harder than normal?	Weekly at noon	Other than your regular job, did you do any physical activities like brisk walking for at least 30 min today?	Change to daily at 8 PM
**Diabetes quality of life**				
	Reply with the number that best describes how much control you felt you had over your diabetes over the past 2 weeks	Biweekly at noon	Retain as is change timing to weekly	Change to weekly at 3 PM
**Emotional health**				
	Reply with the number that best describes how irritable or moody you felt today owing to your diabetes	Daily at 7 PM	Remove	N/A^c^

^a^PRO: patient-reported outcome.

^b^Text in italics show the changes made to the PRO timing across user testing sessions.

^c^N/A: not applicable.

#### Patients: Personalized Reports

A total of 9 patients provided feedback on the 1-month and 3-month versions of the personalized report: 4 of these patients participated in the user testing (of which 2 were recruited from the focus groups), whereas 5 were naive to the program. Overall, the majority of patients (8/9, 89%) felt the report was easy to read, eye-catching, and comprehensive. There was a strong preference for the 1-month version of the report owing to the larger font size. Patients also felt that receiving the report more frequently would help sustain motivation. Patients preferred layouts that used darker fonts and lighter background colors to help make the text easier to read. All patients viewed the color-coded schema favorably because it helped draw attention to the most important aspects of the report and made the data easy to interpret.

Several patients had difficulty reading the bar graphs of PROs that were collected biweekly (eg, quality of life) and recommended changing the items to weekly measures to be consistent with other PROs. Finally, email was the preferred delivery method, and most patients would share the report with their family and friends (Table 6).

Benefits of using the personalized report for diabetes self-management included being able to analyze how well one is adhering to recommended diabetes behaviors (“being honest with yourself”)*,* providing visual cues to take responsibility for one’s health (“a visual reminder of things I need to do but don’t do and how I can be better”), and providing support to stay on track to be successful with diabetes. In the first round of user testing, 3 critiques of the report included being of greater use to providers than patients, concerns about confidentiality, and being too limited because it did not include tips on how patients can improve unhealthy behaviors. From this feedback, we incorporated motivational text messages and insights into the i-Matter prototype and created a study website that included additional resources. Patients in the second round of testing regarded the inclusion of insight messages as a source of motivation to change their behaviors and to continue responding to text messages to monitor changes in behavior over time.

#### Provider Feedback

Overall, all (n=6) providers thought the report was a good tool to help patients manage their T2D. Similar to patients, they felt that the insight messages were helpful for interpreting the data and prompting behavioral changes. When reviewing the PRO content, providers cautioned that before starting the program, patients would need to be educated on the recommended dietary and physical activity guidelines for diabetic patients and the medications they are currently taking for their T2D to ensure they are reliably answering the questions. On the basis of this feedback, at the baseline visit, trained study staff provide a brief overview of evidence-based guidelines for healthy eating and physical activity for T2D using low-literacy and language-congruent patient education handouts from the American Diabetes Association and review the patient’s current diabetes medication regimen.

**Figure 3:**
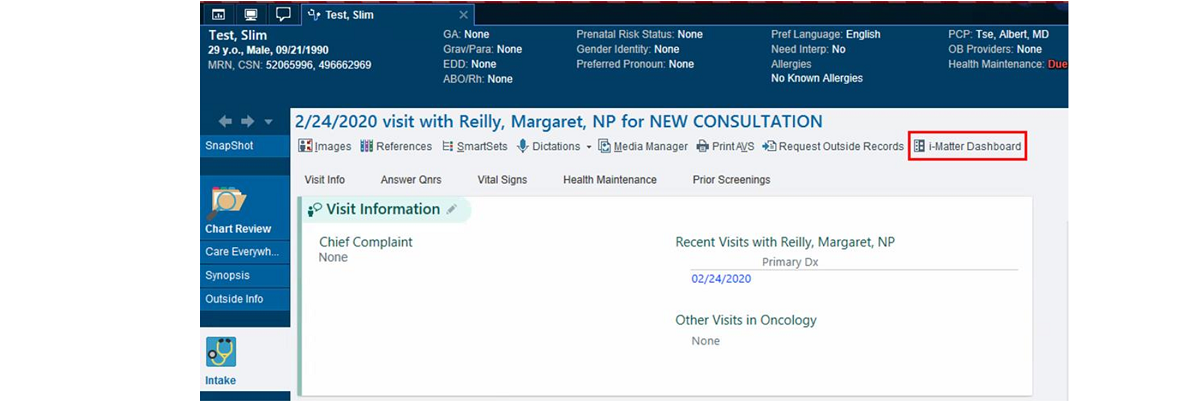
Screenshot of i-Matter Epic integration.

To integrate the report into clinical practice, providers preferred having a separate tab built into the EHR, which included a summary of the personalized report and highlighted key trends in patients’ PRO data over the past 3 months. All providers found value in discussing the report with patients during the clinic visit because the data complemented the questions that they had already asked about diabetes self-management. Finally, although they found value in the longitudinal trends displayed in the graphs, owing to time constraints, they felt that patients should bring up anything important that stood out in the detailed view. On the basis of this feedback, the study team is working in collaboration with NYULH MCIT to integrate the personalized report into Epic. This includes the development of security protocols that will link patients’ encrypted research ID to their medical record number and integrate the report image into an Epic web integration record. Web integration records are used to visually integrate external apps with Epic. Providers will be able to access the i-Matter report via a button located within the patient’s chart at the top of the Office Visit toolbar (Figure 3). The button will only be visible for patients randomized to the intervention arm.

## Discussion

### Strengths

Although achieving glycemic control is of clinical importance, it is the daily experiences of living with T2D that drive patients’ decisions to adhere to treatment recommendations and become engaged in their care [56]. Even with the most efficacious treatments, failure to incorporate patients’ perspective of their disease into clinical decision making will make achieving the outcomes desired by patients and providers unattainable. The i-Matter trial will assess whether a theory-driven mobile PRO system that incorporates a set of PROs that are meaningful to both patients and providers can lead to reductions in HbA_1c_ and improvements in patient adherence to self-care behaviors. Unlike existing programs, i-Matter is designed to collect real-time PRO data in the form of data-driven feedback, motivational messages, and dynamic data visualizations that are displayed in personalized reports for patients and providers.

This paper describes the design and refinement of i-Matter through an iterative user-centered approach that actively involved patients and providers throughout the process. Active involvement of end users in the development of the intervention can help to address the difficulties with protocol compliance, lack of clinical integration in the EHR, and provider skepticism about the utility of PROs in practice, which are hallmarks of previous trials, thus increasing the likelihood of developing a sustainable approach [57]. Findings from our formative phase resulted in several insights regarding issues with the design, usability, and workflow of i-Matter, which led to key changes in the content and delivery of the text messages and personalized report and the technical infrastructure to support the integration of i-Matter into the EHR to improve patient and provider acceptability. In addition to evaluating the clinical benefit of i-Matter, the RCT will provide much needed evidence on the conditions under which mHealth interventions *work* in primary care settings and in patients’ daily lives and the organizational, individual, and technical factors that are required to support their use.

### Limitations

Although there are many strengths of our intervention approach, we note limitations that can be considered for future research. First, although our intervention is designed to target patients with T2D, it is more common for patients to have 2 or more chronic diseases (ie, multimorbidity) than 1 disease in isolation (89.3% vs 8.5%, respectively) [58]. In fact, recent research demonstrates the negative impact of multimorbidity on PROs such as quality life, psychosocial health, self-efficacy, physical function, and self-management behaviors (eg, physical activity and medication adherence) [59]. Thus, future research should examine whether adapting i-Matter for a multimorbid population would improve the integration and coordination of patient and provider management of co-occurring chronic diseases rather than using a single disease focus that can cause inefficiencies and fragmentation in care. Second, we did not perform psychometric testing of the final PROs before they were deployed in our intervention. We will use data collected in this study to assess the psychometric properties of our PRO questions and test their validity in subsequent research.

Finally, 2 (out of the 6) providers interviewed during the development of i-Matter indicated that they found less value in PROs that were not immediately actionable in primary care practice (eg, depression and quality of life). A key strength of the i-Matter study is the full EHR integration of the PROs with the health care team. Many previous PRO initiatives share the patient PRO data back with the providers in a workflow disruptive manner—asking providers to change their normal activities and make a special effort to review the PRO data. i-Matter overcomes these challenges by delivering the patient PRO data directly into the patient’s chart in the EHR—presented as just another commonly viewed data visualization by the provider such as patients’ lab and test results. Thus, our intervention will test the hypothesis that if actionable diabetes PRO data are delivered in the right context, it will influence patient-provider interactions. Early adopters of our intervention will also help to provide important data on the potential effectiveness and (time) efficiency of using PROs in clinical care. Sharing the outcomes of this work could provide providers who are hesitant to adopt such innovations with much needed information about the benefits of using these tools.

## Appendix

Multimedia Appendix 1 Example motivational and feedback messages.

## Abbreviations

COM-B: capability, opportunity, and motivation model of behavior
EHR: electronic health record
ESRD: end-stage renal disease
HbA1c: hemoglobin A1c
i-Matter: investigating an mHealth texting tool for embedding patient-reported data into diabetes management
MCIT: Medical Center Information Technology
mHealth: mobile health
NYULH: New York University Langone Health
PRO: patient-reported outcome
RA: research assistant
RCT: randomized controlled trial
T2D: type 2 diabetes
TAM: technology acceptance model
UCD: user-centered design
