# Supplementary material for: Investigation of a Mobile Health Texting Tool for Embedding Patient-Reported Data Into Diabetes Management (i-Matter): Development and Usability Study
Source: JMIR Form Res. 2020 Aug 31;4(8):e18554. doi: 10.2196/18554 (PMC7490676; doi:10.2196/18554)
Supplement: Multimedia Appendix 1 [file formative_v4i8e18554_app1.docx]

**Example Motivational and Feedback Messages**

| **Motivational Messages** | | **English Version** | **Spanish Version** |
| --- | --- | --- | --- |
| **Completion-based** | | | |
| **Week 1** | | i-Matter (TM): Thank you for completing the first week. Your willingness to participate in the i-Matter program is appreciated. | i-Matter (TM): Gracias por completar la primera semana. Agradecemos su disposición de participar en el programa i-Matter. |
| **Month 1** | | i-Matter (TM): You are off to a great start. Keep putting in the work to better manage your diabetes and commit to a better you! | i-Matter (TM): Usted ha tenido un gran comienzo. ¡Siga esforzándose para controlar mejor su diabetes y disponerse a mejorarse así mism@! |
| **Month 3** | | i-Matter (TM): You have finished 25% of the i-Matter program. We appreciate your efforts so far! | i-Matter (TM): Usted ha terminado el 25% del programa i-Matter. Agradecemos sus esfuerzos hasta el momento! |
| **Response-based** | | | |
| **Sleep Quality PRO** | i-Matter (TM): Practicing a relaxing bedtime ritual can be a way to get sound and deep sleep. Stay away from bright lights and activities that induce stress. | | i-Matter (TM): Practicar una rutina de meditación puede ayudarle a dormir sana y profundamente. Evite las luces brillantes y las actividades que inducen estrés. |
| **Diabetes Quality of Life PRO** | i-Matter (TM): Your participation in the i-Matter study helps to manage your diabetes & also can lead to overall improved health. Chose to stay healthy for you! | | i-Matter (TM): Su participación en el estudio i-Matter le puede ayudar a controlar su diabetes y mejorar su salud en general. ¡Elija mantenerse en salud! |
| **Activity-based** | | | |
| **Active** | i-Matter (TM): You’ve responded to XX% of messages over the last week! | | i-Matter (TM): ¡Usted ha respondido al XX% de los mensajes enviados en la última semana! |
| **Inactive** | i-Matter (TM): It can be hard to consistently respond to your i-Matter messages. Creating a reminder system can help. | | i-Matter (TM): Puede ser difícil responder constantemente a sus mensajes del programa i-Matter. Crear un sistema de notificación puede ayudarle a responder. |

| **Insight Messages** | **English Version** | **Spanish Version** |
| --- | --- | --- |
| **Combination** | | |
| **Sleep Quality + Physical Activity** | On weeks you report good sleep, you also report being physically active. | Cuando informa que duerme bien, también indica que está físicamente activo/a. |
| **Medication Adherence + Quality of Life** | When you report taking your medication every day you also feel on top of your diabetes. | Cuando informa que toma su medicamento todos los días, usted también se siente en control sobre su diabetes. |
| **Individual** | | |
| **Positive Trend** | On week X you reported a high score of X for [0-10 PRO]. Make it a habit! | En la semana X indicó un récord de X en su [0-10 PRO]. ¡Hágalo un hábito! |
| **Negative Trend** | You had X for [0-10 PRO] on average this month. Start today. What can you do differently? | En este mes usted tuvo un promedio de X en su [0-10 PRO]. Empiece hoy, ¿qué puede hacer diferente? |
